# Successful Regulatory T Cell-Based Therapy Relies on Inhibition of T Cell Effector Function and Enrichment of FOXP3+ Cells in a Humanized Mouse Model of Skin Inflammation

**DOI:** 10.1155/2020/7680131

**Published:** 2020-05-14

**Authors:** S. Landman, V. L. de Oliveira, M. Peppelman, E. Fasse, E. van Rijssen, S. C. Bauland, P. van Erp, I. Joosten, H. J. P. M. Koenen

**Affiliations:** ^1^Laboratory of Medical Immunology, Department of Laboratory Medicine, Radboud University Medical Center, Radboudumc. P.O. Box 9101, 6500 HB, Nijmegen, Netherlands; ^2^Department of Dermatology, Radboud University Medical Center, Radboudumc. P.O. Box 9101, 6500 HB, Nijmegen, Netherlands; ^3^Bauland Kliniek, Mill, Netherlands

## Abstract

**Background:**

Recent clinical trials using regulatory T cells (Treg) support the therapeutic potential of Treg-based therapy in transplantation and autoinflammatory diseases. Despite these clinical successes, the effect of Treg on inflamed tissues, as well as their impact on immune effector function *in vivo*, is poorly understood. Therefore, we here evaluated the effect of human Treg injection on cutaneous inflammatory processes *in vivo* using a humanized mouse model of human skin inflammation (huPBL-SCID-huSkin).

**Methods:**

SCID beige mice were transplanted with human skin followed by intraperitoneal (IP) injection of 20‐40 × 10^6^ allogeneic human PBMCs. This typically results in human skin inflammation as indicated by epidermal thickening (hyperkeratosis) and changes in dermal inflammatory markers such as the antimicrobial peptide hBD2 and epidermal barrier cytokeratins K10 and K16, as well as T cell infiltration in the dermis. *Ex vivo*-expanded human Treg were infused intraperitoneally. Human cutaneous inflammation and systemic immune responses were analysed by immunohistochemistry and flow cytometry.

**Results:**

We confirmed that human Treg injection inhibits skin inflammation and the influx of effector T cells. As a novel finding, we demonstrate that human Treg injection led to a reduction of IL-17-secreting cells while promoting a relative increase in immunosuppressive FOXP3+ Treg in the human skin, indicating active immune regulation in controlling the local proinflammatory response. Consistent with the local control (skin), systemically (splenocytes), we observed that Treg injection led to lower frequencies of IFN*γ* and IL-17A-expressing human T cells, while a trend towards enrichment of FOXP3+ Treg was observed.

**Conclusion:**

Taken together, we demonstrate that inhibition of skin inflammation by Treg infusion, next to a reduction of infiltrating effector T cells, is mediated by restoring both the local and systemic balance between cytokine-producing effector T cells and immunoregulatory T cells. This work furthers our understanding of Treg-based immunotherapy.

## 1. Introduction

Regulatory T cells (Treg) play a central role in immune homeostasis and prevention of autoimmune diseases [1–3]. In a variety of transplantation and autoimmune mouse models, injection of Treg prevented immune pathology [4–7]. Promising clinical effects of Treg therapy with expanded Treg in the treatment of patients with graft versus host disease (GvHD) have been demonstrated [8–10]. Moreover, Treg therapy is currently tested in solid organ transplantation [7, 11, 12]. In living donor liver transplantation, a pilot study with *ex vivo*-expanded Treg was shown to be effective and to induce operational tolerance [13]. Also, in autoimmunity and chronic inflammatory diseases, Treg therapy is studied with the aim to control the disturbed immune balance. Already promising results were obtained in small-scale phase I/II clinical trials in type 1 diabetes (T1DM) [14, 15], Crohn's disease [16], and uveitis [17]. As Treg are dysfunctional in psoriatic patients [18, 19], Treg-based therapy might be an interesting treatment option for patients with severe psoriasis.

Treg-based therapy could reduce toxicity and side effect in comparison with immunosuppressive drugs. The ultimate goal of Treg therapy would be induction of tolerance without immune suppression-related development of malignancies and infections [4, 5, 20]. Despite the current successes of Treg-based therapy, it is still not precisely known how and where immune regulation by injected Treg take place and in which anatomical side the inflammatory processes are controlled. Studies in mouse models have already shown that Treg often accumulate at the site of inflammation, most likely to control the duration and the extent of inflammation and in doing so protect the host from immune-mediated pathologies [12–14]. Physiological trafficking and migration to tissues and secondary lymphoid organs are crucial for Treg suppression functions *in vivo* [21].

Humanized mouse models (i.e., immune-deficient mice equipped with both human tissue and a competent human immune system) provide a useful *in vivo* preclinical tool for assessment of the human immune system and its influence on inflammatory processes of human tissue [22, 23]. However, reports characterizing the immune responses after Treg therapy are scarce [8–10, 24–27].

Here, we used the huPBL-SCID-huSkin allograft model [28], which enables quantitative analysis of the human dermal inflammatory response and the systemic immune response *in vivo* [29], to study the effect of human Treg infusion. We here demonstrate that normalization of the inflammatory skin response by Treg injection, next to inhibiting T cell infiltration, is the result of both local and systemic immunosuppression of T cell-mediated effector cytokine production as well as fostering a relative increase in immunosuppressive FOXP3+ Treg in the skin.

## 2. Materials and Methods

### 2.1. Mice

The huPBL-SCID-huSkin allograft model used in this study is described in detail by de Oliveira et al. [29]. Female B17.B6-Prkdc^scid^Lyst^bg/Crl^ (SCID beige) mice, 6-8 weeks old (Charles River Breeding Laboratories), were transplanted with human skin from healthy individuals obtained after abdominal plastic surgery at Bauland Kliniek (Mill, Netherlands). After healing of the human skin (21 days), 2‐4 × 10^7^ (depending on the available cell numbers) peripheral blood mononuclear cells (PBMCs) were injected intraperitoneally (IP) in the absence or presence of an equal number of Treg (ratio of 1 : 1, PBMC : Treg).

The experiments in our current study were performed using 3 series of experiments using the skin from 3 different skin donors. Every series of experiments consisted of 3 groups: a PBS group, a human PBMC group, and a human PBMC+expanded Treg group. The number of experiments that could be performed was dependent on the numbers of human cells that were obtained from the buffy coats and following Treg expansion, resulting in 2–5 animals per group; overall, the PBS, PBMC, and PBMC+Treg consisted of *n* = 6, *n* = 13, and *n* = 8 mice. Unless stated otherwise, these numbers were used for analysis. All animal experimental procedures were in accordance with the international welfare guidelines and approved by the institutional animal ethical committee of the Radboud University in Nijmegen (DEC 2010-153). Mice were sacrificed 3 weeks after cell injection by cervical dislocation.

### 2.2. Human Materials

The use of human skin and peripheral blood was approved and in accordance with the regulations set by the Medical Ethical Committee for human research of the Radboudumc. Human skin and buffy coats (Sanquin Blood Bank, Nijmegen, Netherlands) were obtained from healthy donors, who gave written consent for scientific use according to the Declaration of Helsinki. All experiments were performed in accordance with relevant guidelines and regulations.

### 2.3. Cell Isolation and Regulatory T Cell Expansion

Human PBMC were isolated by Ficoll density gradient separation (Lymphoprep, Nycomed-Pharma AS, Norway) of buffy coats. Approximately 200 × 10^6^ PBMCs were stored in liquid nitrogen, and from the remaining PBMC, CD4+ cells were isolated by negative selection using MACS anti-CD4 microbeads according to the manufacturer's instructions. Thereafter, CD25+ cells were isolated by positive selection, using magnetic separation by MACS anti-CD25 microbeads (Miltenyi Biotec, Germany) combined with a MS column and a Vario MACS magnetic cell sorter (Miltenyi, Biotec, Germany) according to the manufacturer's instructions. This typically resulted in >90% pure Treg, based on FOXP3 expression. Isolated CD4+CD25+ cells were expanded for 7 days *in vitro* by stimulation with *α*CD3/*α*CD28 stimulator beads (Invitrogen, United Kingdom) in a 1 : 2 bead to cell ratio in the presence of 250 U/ml IL-2 (Proleukin (Alloga, United Kingdom)). A second expansion round of 6 days was performed with *α*CD3/*α*CD28 beads in a 1 : 4 bead to cell ratio in the presence of 1000 U/ml IL-2. Before injection, the expanded Treg were allowed to recover for one day in medium+IL-2 (200 U/ml). Thawed PBMC and expanded Treg were injected IP in a ratio of 1 : 1 in PBS.

### 2.4. *In Vitro* Suppression Assay

The *in vitro* suppressive function of the expanded human Treg was assessed in a coculture suppression assay. To this end, autologous CD4+CD25- cells (25 × 10^3^) were stimulated with *α*CD3/*α*CD28 stimulator beads in a 1 : 5 bead to cell ratio and cocultured with grading numbers of expanded Treg. Titration of the CD4+CD25- cells was included for control purposes. Proliferation was measured at day 5 by an addition of 0.5 *μ*Ci ^3^H-Thymidine (Amersham Biosciences, Piscataway, NJ) for at least 6 h. Tests were set up in triplicate. Based on the ^3^H-Thymidine data, the percentage inhibition was calculated as mean percentage inhibition ± SEM of 8 independent experiments performed with cells from different donors as shown.

### 2.5. Flow Cytometry and Antibodies

Cell phenotype was analysed by a multicolor flow cytometer Navios (Beckman-Coulter, Mijdrecht, Netherlands). For surface staining, the following anti-human conjugated monoclonal antibodies were used: CD3-(UCHT1) ECD, CD4-(13B8.2) PC5.5, CD45-(J33) KO, CD25-(MA251) PECY7 BD, CD8-(B9.11) APC700, or APC750 (all from Beckman-Coulter). In addition, the following anti-human conjugated monoclonal antibodies were used for intracellular and cytokine measurements: IFN-*γ*-(4S.B3) PECY7, IL17A-(eBio64DEC17) APC-e780, and FOXP3-(PCH101) PB (all from eBioscience) and Ki67 (B56) Alexa-Fluor 488 (BD Bioscience). Intracellular analysis was performed after fixation and permeabilization, using a Fix and Perm reagent (eBioscience) according to the manufacturer's instructions. Before intracellular cytokine measurements, the cells were stimulated for 5 h with PMA (12.5 ng/ml), ionomycin (500 ng/ml), and Brefeldin A (5 *μ*g/ml) (all from Sigma-Aldrich). Fluorescence minus one (FMO) stainings were used to control and determine gate settings.

### 2.6. Histology and Immunohistochemistry

Human skin grafts were fixed in neutral buffered 4% formalin (Mallinckrodt Baker Inc., Deventer, Netherlands) for 4 h, processed, and embedded in paraffin. Then, 6 *μ*m sections were cut and the slides were stained with hematoxylin-eosin (HE) or processed for immunohistochemical staining. The following human monoclonal antibodies were used: anti-CD3 (clone 7.2.38, Abcam, Cambridge, UK), anti-FOXP3 (PCH101, eBioscience), anti-Keratin-10 (K10, RKSE60; euro-diagnostica), anti-Keratin-16 (K16, LL025; Monosan), anti-*β*-defensin-2 (hBD2, Abcam, Cambridge, UK), anti-CD4 (Santa Cruz BC/F6), anti-CD8 (144B DAKO) and anti-IL-17 (Polyclonal goat IgG, R&D Systems). To detect K10, K16, hBD2, CD4, and CD8, the sections were incubated with EnVision labeled polymer anti-mouse (DAKO) and visualized using 3,3′-diaminobenzidine (DAB). To detect CD3, FOXP3, and IL-17, sections were stained using the Labeled Streptavidin Biotin method (Universal LSAB+ Kit/AP, DAKO) and visualized using Permanent Red (DAKO). Antibody stainings were visualized using the Dako Cytomation EnVision+ System-HRP (ABC) kit (DAKO, Glostrup, Copenhagen, Denmark) combined with 3,3′-diaminobenzidine tetrahydrochloride (DAB, brown, Sigma-Aldrich). Immunohistochemistry control staining was conducted by omitting the primary antibody staining step. In the absence of primary antibodies, no staining was detected (not shown). Sections were photographed at the indicated magnification using a microscope (Axioskop 2 MOT; Zeiss) and a digital camera (Axiocam MRc5; Zeiss) and AxioVision software (Zeiss).

### 2.7. Immunohistochemistry Quantification and Determination of Epidermal Thickness

To analyse human CD4 and CD8 cells, representative pictures were taken at 10x magnification and pictures were analysed using ImageJ software. A representative region of interest (ROI) was drawn from the lowest epidermal papilla till 300 *μ*m depth into the dermis. Cell quantification was performed by setting a threshold and relating this to a number of cells per mm^2^. The total epidermal area and K10, K16, and hBD2-positive area were measured using ImageJ in the region of interest (ROI) and displayed as %K10, %K16, or %hBD2-positive epidermal area. The average epidermal thickness (*μ*m) was calculated by taking the mean of multiple thick and thin parts of the epidermis (using ImageJ).

### 2.8. Statistical Analysis

The results were statistically analysed by a one-tailed Mann–Whitney *U* test using GraphPad Prism software version 5.03. Differences with a *p* value of <0.05 were considered significant and are indicated with an asterisk (^∗^): *p* value < 0.01 (^∗∗^), *p* value < 0.001 (^∗∗∗^). The experimental groups were blinded for the animal caretakers and technicians who analysed the animals and tissues. This information was only available to the responsible researcher. Unless stated otherwise, all results are biological replicates.

## 3. Results

### 3.1. Suppression of Human Skin Inflammation by *Ex Vivo*-Expanded Human CD4+ Regulatory T Cells

**Figure 1:**
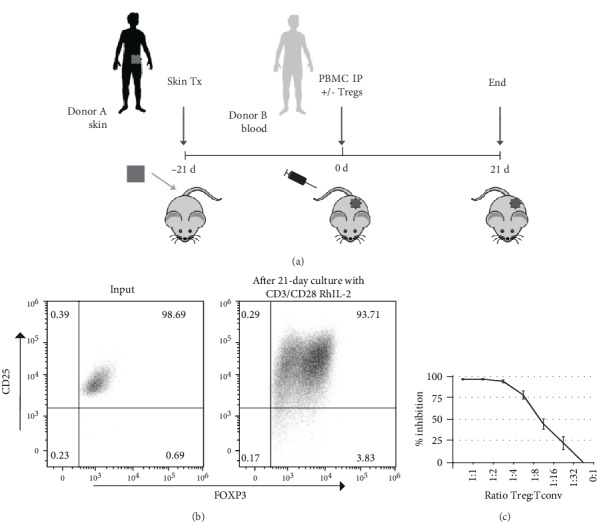
Expanded human Treg maintain their suppressive capacity *in vitro*. (a) Schematic representation of the huPBL-SCID-huSkin allograft model with adoptive transfer of PBMC combined with or without Treg (1 : 1 ratio). (b) Flow cytometric analysis of Treg before and after expansion. Representative dot plot showing CD25 and FOXP3 expression of input cells and expanded human Treg, respectively. (c) Suppressive capacity of the *ex vivo*-expanded Treg was examined using an *in vitro* suppression assay. The graph shows the Treg : Tconv ratio (*x*-axis) and percentage inhibition of Tconv proliferation (*y*-axis) as analysed by ^3^H-Thymidin incorporation. Mean ± SEM is shown, *n* = 8.

**Figure 2:**
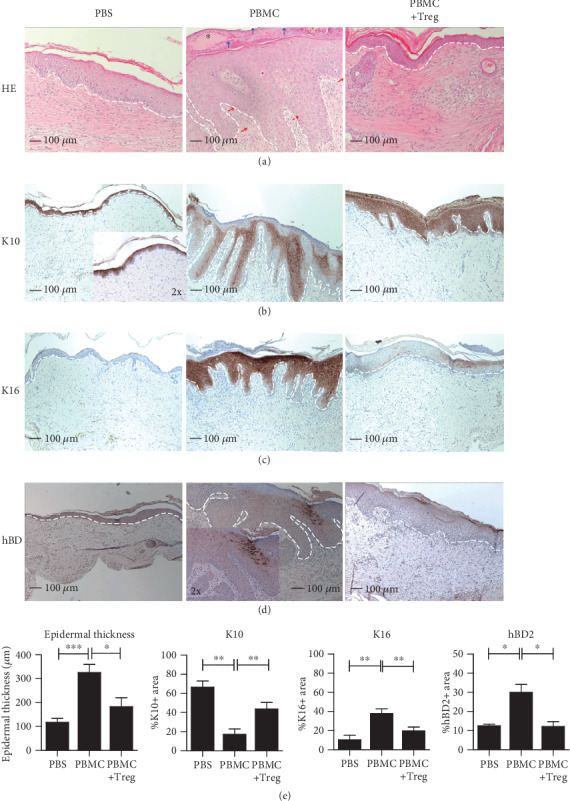
Treg infusion inhibits PBMC-induced skin inflammation *in vivo*. Representative histology at 10x magnification and quantitative analysis of human skin grafts from SCID beige mice at 21 days after cell injection of PBMC with or without *ex vivo*-expanded Treg. The white line indicates the border of the epidermis. The inlay at (b) PBS and (d) PBMC shows 20x magnification: (a) epidermal thickness (*μ*m, HE staining). Parakeratosis is pointed by blue arrows and human cell infiltration by red arrows, and a microabscess is indicated by an asterisk. (b–d) Epidermal expression patterns of K10, K16, and hBD2. (e) Bar plots show the mean ± SEM percentages of the positive area of (a–d), and (b–d) show the percentage of positive cells within the epidermis. PBS (*n* = 6), PBMC (*n* = 13), and PBMC+Treg (*n* = 8). Statistical significance was analysed by the Mann–Whitney *U* test. ^∗^*p* value < 0.05, ^∗∗^*p* value < 0.01, and ^∗∗∗^*p* value < 0.001.

To evaluate the functional suppressive capacity of *ex vivo*-expanded Treg *in vivo*, we have used the huPBL-SCID-huSkin allograft model. The experimental protocol is visualized in Figure 1(a). Human peripheral blood CD4+CD25+ Treg were isolated by positive isolation using magnetic beads. The isolated CD4+CD25+ cells were 94-98.9% pure as indicated by coexpression of CD25 and FOXP3 (Figure 1(b)). These isolated CD4+ Treg were expanded *ex vivo* in two subsequent expansion cycles using *α*CD3/*α*CD28 bead stimulation in the presence of exogenously added human recombinant IL-2. This resulted in a more than 100-fold expansion in cell number (data not shown). After expansion, the cells retained expression of CD4, CD25, and FOXP3 (Figure 1(b)) and revealed potent suppressive capacity *in vitro* (Figure 1(c)). As expected, injection of PBMC alone led to inflammation of the transplanted human skin as indicated by a clear increase in the epidermal thickness of the human skin. Coinjection of human *ex vivo*-expanded Treg, at a PBMC : Treg ratio of 1 : 1, successfully suppressed cutaneous inflammation induced by allogeneic PBMC, and prevented epidermal thickening. (Thickness PBS = 66.61 *μ*m ± 6.25, PBMC = 325.3 *μ*m ± 33.98, and PBMC + Treg = 182.8 *μ*m ± 37.36. PBS vs. PBMC: *p* = 0.0001, PBMC vs. Treg: *p* = 0.0156.) (Figure 2(a)). To study the effect of Treg coinjection on the dermal inflammatory response as induced by PBMC injection, we analysed the expression of the skin pathology-related proteins Keratin-10 (K10) (PBS = 66.61% ± 6.25, PBMC = 17.18% ± 5.62, and PBMC + Treg = 43.81% ± 6.60. PBS vs. PBMC: *p* = 0.0016, PBMC vs. PBMC+Treg: *p* = 0.0055) (Figure 2(b)), Keratin-16 (K16) (PBS = 10.59% ± 4.52, PBMC = 37.86% ± 4.98, and PBMC + Treg = 19.90% ± 3.81. PBS vs. PBMC: *p* = 0.0028, PBMC vs. PBMC+Treg: *p* = 0.0056) (Figure 2(c)), and human *β*-defensin-2 (hBD2) (PBS = 12.50% ± 0.87, PBMC = 29.98% ± 4.10, and PBMC + Treg = 12.15% ± 2.60. PBS vs. PBMC: *p* = 0.0104, PBMC vs. PBMC+Treg: *p* = 0.0106) (Figure 2(d)) using immunohistochemistry. As reported previously [15], injection of PBMC led to downregulation of K10 and a parallel induction of K16 and hBD2 expression (Figures 2(b)–2(d)). Here, we demonstrate that coinjection of Treg inhibited K10 downregulation as well as the induction of K16 and hBD2 (Figures 2(b)–2 (d)) and thus normalizes epidermal inflammation.

### 3.2. Human Treg Injection Inhibits Skin Inflammation by Reducing Effector T Cell Infiltration and Enrichment of FOXP3+ Treg

**Figure 3:**
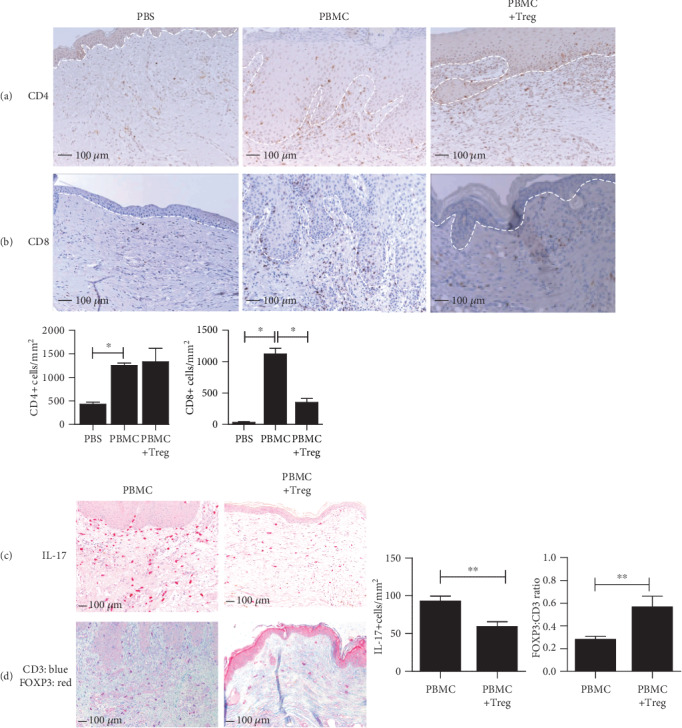
Treg infusion inhibits skin-infiltrating T cells and downregulates local IL-17 production while promoting FOXP3+ Treg enrichment. Representative histology at 10x magnification and quantitative analysis in skin grafts from SCID beige mice at 28 days after cell injection. Expression of human (a) CD4+ and (b) CD8+ T cells. (c) Expression of IL-17-secreting cells and (d) expression of CD3+ (blue) and FOXP3 (red). Mean ± SEM is shown for (a–c) (*n* = 3). Statistical significance was analysed by the Mann–Whitney *U* test. ^∗^*p* value < 0.05; ^∗∗^*p* value < 0.01.

As previously shown [29], following injection of PBMC human CD4+ and CD8+, T cells can be detected in the skin of the human skin transplant and secondary organs. Coinjection of *ex vivo*-expanded Treg significantly inhibited accumulation of human CD8+ T cell (PBMC = 1121 cells/mm^2^ ± 91.80, PBMC + Treg = 349.8 cells/mm^2^ ± 61.18, *p* = 0.0383) in both epidermis and dermis while CD4+ T cell numbers (PBMC = 1253 cells/mm^2^ ± 50.28, PBMC + Treg = 1329 cells/mm^2^ ± 287.8, NS) remained unchanged (Figures 3(a) and 3(b)). Injection of PBMC in the huPBL-SCID-huSkin model typically results in human skin-infiltrating IL-17+ cells [29]. Coinjection of *ex vivo*-expanded human Treg led to a significant inhibition of IL-17+ cells (PBMC = 92.96 cells/mm^2^ ± 6.56, PBMC + Treg = 59.10 cells/mm^2^ ± 6.39, *p* = 0.032) (Figure 3(c)). Coinjection of Treg led to a relative increase of human FOXP3+ cells in the transplanted human skin, as indicated by the significantly increased FOXP3+ : CD3+ T cell ratio (PBMC = 0.28 ± 0.012, PBMC + Treg = 0.5675 ± 0.04732, *p* = 0.0097) (Figure 3(d)).

### 3.3. Treg Injection Affects Circulating Human T Cell Numbers and Their Proinflammatory Cytokine-Producing Potential

**Figure 4:**
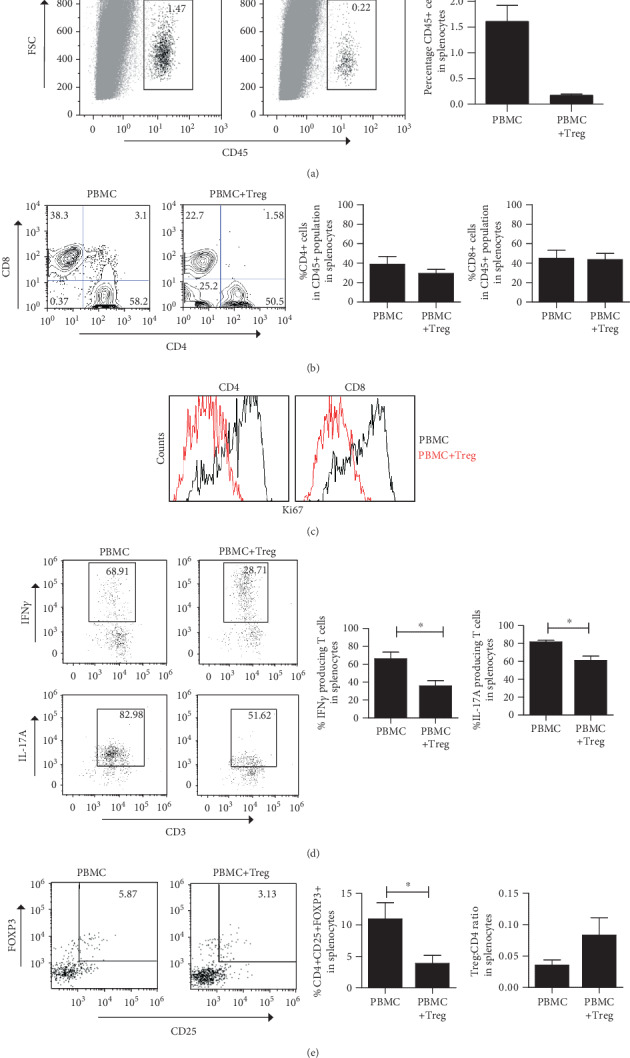
Treg infusion affects systemic proinflammatory cytokine production by T cells. Representative flow cytometry pictures and quantitative analysis of systemic human CD45+ and CD3+ T cells harvested from the mouse spleen of SCID beige mice infused with PBMC with or without Treg. (a) Percentage of human CD45 cells. (b) Percentage of human CD4+ and CD8+ cells within human CD45+ cells. (c) Representative example of the percentages of dividing (Ki67+) CD4+ and CD8+ cells (*n* = 3). (d) Percentage of human IFN*γ* and IL-17A-secreting T cells. (e) Frequency of CD4+CD25+FOXP3+ cells within CD45+ cells and Treg : CD4 ratio analysis. Mean ± SEM is shown for (a, b, d) (*n* = 3‐8). Statistical significance was analysed by the Mann–Whitney *U* test. ^∗^*p* value < 0.05; ^∗∗^*p* value < 0.01.

Injection of PBMC results in repopulation of human CD4+ and CD8+ T cells in the SCID beige mice. Coinjection of *ex vivo*-expanded human Treg inhibits this repopulation as indicated by a strong reduction of T cells in the spleen (PBMC = 1.61% ± 0.31, PBMC + Treg = 0.17% ± 0.03, *p* = 0.0286) (Figure 4(a)). However, similar CD4+ (PBMC = 38.77% ± 8.19, PBMC vs. Treg 29.38% ± 4.07, NS) and CD8+ (PBMC = 45.02% ± 8.28, PBMC + Treg = 43.46% ± 6.45, NS) percentages were observed in the spleen in the absence and presence of injected Treg (Figure 4(b)). Inhibition of CD4+ and CD8+ T cell proliferation by coinjected Treg was supported by Ki67 staining of CD4+ and CD8+ cells (Figure 4(c)**)** Notably, Treg coinjection led to a significant reduction of IL-17A (PBMC = 81.65% ± 1.91, PMBC + Treg = 60.97% ± 4.81, *p* = 0.0242) as well as IFN*γ* (PBMC = 66.01% ± 7.43, PBMC + Treg = 35.78% ± 5.85, *p* = 0.0485) expressing human CD4+ T cells in the mouse spleen (Figure 4(d)), while a trend towards an increase in the relative numbers of FOXP3+ cells was observed (PBMC = 0.0357 ± 0.0081, PBMC + Treg = 0.0833 ± 0.0277, *p* = 0.1101) (Figure 4(e)).

## 4. Discussion

Humanized mouse models offer the opportunity to study the human immune response *in vivo* and to perform preclinical immune intervention studies [22, 23, 28, 30, 31]. The huPBL-SCID-huSkin allograft model allows to study skin inflammation and intervention of T cell-driven skin inflammation [29]. Here, we used this huPBL-SCID-huSkin allograft model to study the *in vivo* suppressive potential of *ex vivo*-expanded human Treg on skin inflammation thereby focusing on regulation of dermal inflammatory markers and on effector cytokine expression by T cells.

It has been demonstrated that Treg injection in humanized mouse models prevents expansion of the human T cells. In the presented work, we elaborate further on the immunosuppressive mechanism of Treg *in vivo* by demonstrating that inhibition of skin inflammation by the injection of *ex vivo*-expanded Treg is mediated by inhibition of cytokine effector T cell function and enrichment of FOXP3+ Treg. Treg injection inhibited epidermal thickening and restored inflammation-related aberrant epidermal expression of K10/K16 and hBD2 expression. This Treg-mediated dermal restoration is most likely explained by both local and systemic effects. Systemically, in the spleen, Treg injection led to reduction of the absolute numbers of circulating T cells and these T cells revealed a reduced potential to produce INF*γ* and IL-17. Additionally, a trend towards increased FOXP3 : CD4 ratio was observed. Locally, in the skin, a reduced CD8+ T cell influx was observed that was paralleled by a relative increase of FOXP3+ regulatory T cells and reduced numbers of IL-17 expressing cells. Thus, Treg injection promotes immune homeostasis in our humanized mouse model. This is in line with findings of others that studied Treg infusion in alternative humanized mouse models [26, 32, 33].

Inflammatory cytokines like IL-17 and IFN*γ* play a key role in the pathology of (auto)inflammatory diseases [34]. In our current work, we demonstrate that Treg injection inhibits the expression of the proinflammatory cytokines IFN*γ* and IL-17 by T cells *in vivo*. IFN*γ* is a cytokine involved in T helper-1-driven immune responses inducing inflammatory responses and apoptotic cell death. T cell-derived IFN*γ* is one of the most potent activators of the proinflammatory functions of keratinocytes, resulting in the expression of a wide array of chemokines, cytokines, and membrane molecules that orchestrate the recruitment, activation, and retention of specific leukocyte subpopulations in the skin [35]. IL-17 is important for host defense but has also been associated with chronic inflammation and autoimmunity [36]. The proinflammatory effect of IL-17 is further potentiated when IFN*γ* is also present [19]. Both IFN*γ* and IL-17 promote expression of the skin antimicrobial peptide hBD2 [18]. hBD2 promotes the recruitment of a variety of immune cells by interacting with chemokine receptors such as CCR6 [37]. The mutual activation of T lymphocytes and keratinocytes has a primary role in the amplification of skin inflammation during immune-mediated skin diseases [20]. In our humanized mouse model, we demonstrate that the peripheral human T cells express both IFN*γ* and IL-17 and we found a parallel increase of epidermal hBD2 expression. Injection of *ex vivo*-expanded Treg led to a reduction in the numbers of circulating human T cells that reveal reduced expression of IFN*γ* and IL-17A as well as reduced hBD2 expression by the keratinocytes. Together, this suggests that inhibition of skin inflammation by Treg injection results in increased Treg in the skin and reduced proinflammatory cytokine production of IFN*γ* and IL-17A by the human T cells, which in turn results in normalization of epidermal marker expression and prevention of epidermal thickening.

In our humanized model, we have been studying Treg : PBMC ratios of 1 : 1, which cannot be considered as physiological ratios. In other humanized mouse models, Treg : PBMC ratios of 1 : 5/1 : 10 were required to inhibit the inflammatory response against islet [38], arterial [27], and skin [29, 39] allografts. In a similar humanized skin allograft BALB/c Rag2−/−c*γ*−/− model, it has been demonstrated that at a Treg : PBMC ratio of 1 : 10, the immunosuppressive effect of Treg infusion was lost [40]. Using our humanized mouse allograft SCID/beige model, similar observations were made. Interestingly, intradermal Treg injection at Treg : PBMC ratios of 1 : 400 [31] still resulted in the inhibition of skin inflammation revealing that alternative administration routes should be considered for clinical application of Treg-based therapy.

Administration of Treg resulted in both systemic and local (i.e., human inflamed skin) immune suppression as indicated by the reduced influx of human T cells in the human-transplanted skin and the reduced presence of human T cells in the spleen. In addition, Treg injection led to a clear local enrichment of FOXP3+ Treg in the transplanted human, and we observed a trend towards an increase of FOXP3+ cells in the periphery. Probably, the infused Treg expand in the draining lymph nodes and migrate specifically to the skin. It has been shown in humans that lymph nodes facilitate Treg expansions [41] and that homing marker expression of cutaneous leukocyte antigen (CLA) on Treg promotes Treg migration to the human skin [40]. Dynamic Treg tracking experiments are needed to further understand their migratory route and clarify the specific site of action of injected Treg. This knowledge and a better understanding of how chemokines and integrins control the migration and survival of distinct Treg subsets might enable selection of Treg subsets with defined homing potential that can be applied to specifically target inflamed tissues such as in Crohn's disease, psoriasis, and transplantation [40, 42].

In conclusion, we here demonstrate that inhibition of skin inflammation by the injection of *ex vivo*-expanded human Treg *in vivo* in a humanized mouse model, next to reducing the influx of T cells, depends on inhibition of effector cytokine production by T cells and enrichment of FOXP3+ regulatory T cells. Altogether, this prevents dermal inflammatory pathology. These results further support the use of Treg-based cell therapy.

## Data Availability

The primary data used to support the findings of this study are available from the corresponding author upon request.

## Abbreviations

K10: Keratin-10
K16: Keratin-16
hBD2: Human *β*-defensin 2
SCID mice: Severe combined immune-deficient mice
PBMC: Peripheral blood mononuclear cells
Treg: Regulatory T cells
IP: Intraperitoneal.

## Supplementary Materials

Supplementary Materials Immunohistochemistry staining for K16 in the human inflamed skin of humanized mice infused with human PBMC. Sections were stained in the absence or presence of a primary antibody directed against K16 followed by a secondary antibody staining and visualization step.
