# Supplementary figures and images for: Successful Regulatory T Cell-Based Therapy Relies on Inhibition of T Cell Effector Function and Enrichment of FOXP3+ Cells in a Humanized Mouse Model of Skin Inflammation
Source: J Immunol Res. 2020 May 14;2020:7680131. doi: 10.1155/2020/7680131 (PMC7244960; doi:10.1155/2020/7680131)

539 **Supplemental figure**  
540

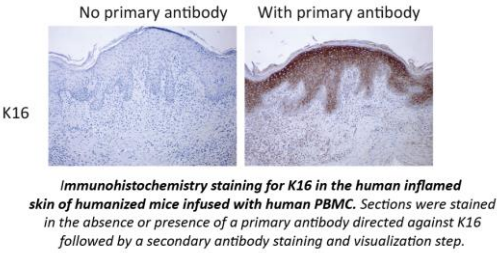

541

Supplement: Supplementary Materials — Immunohistochemistry staining for K16 in the human inflamed skin of humanized mice infused with human PBMC. Sections were stained in the absence or presence of a primary antibody directed against K16 followed by a secondary antibody staining and visualization step. [file 7680131.f1.pdf]
